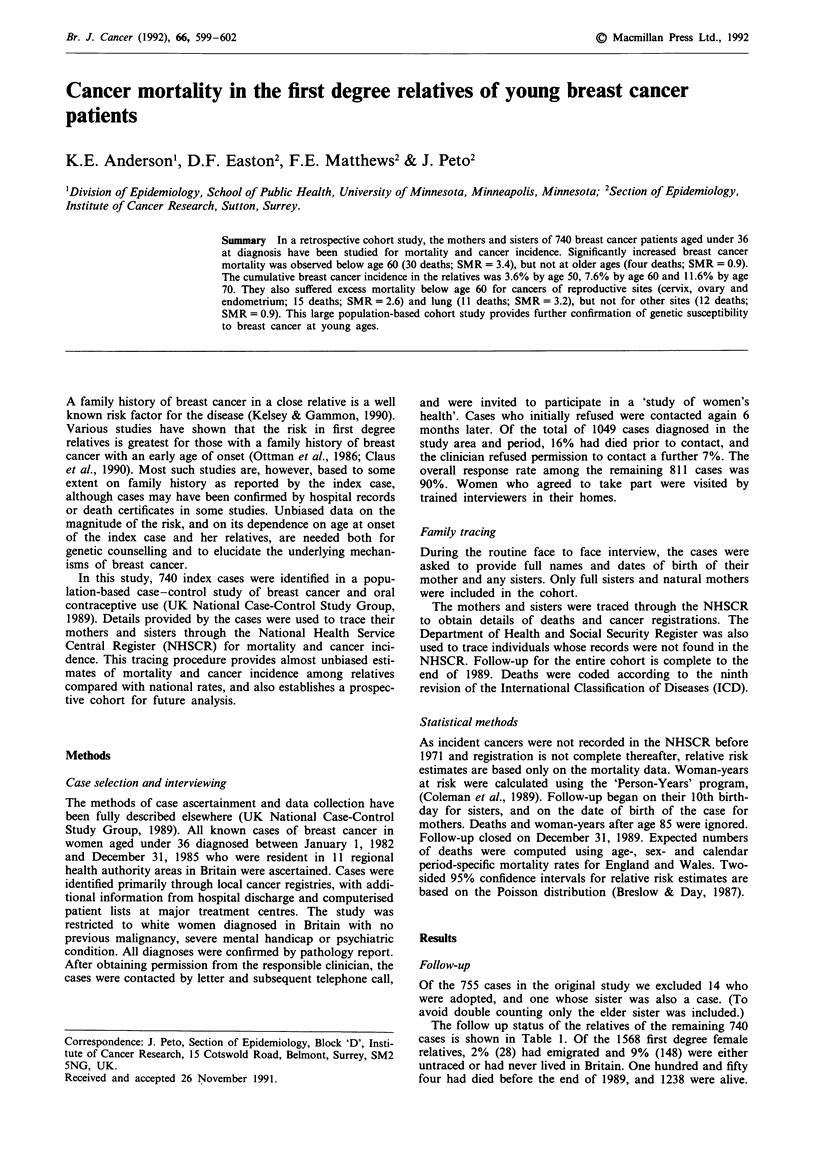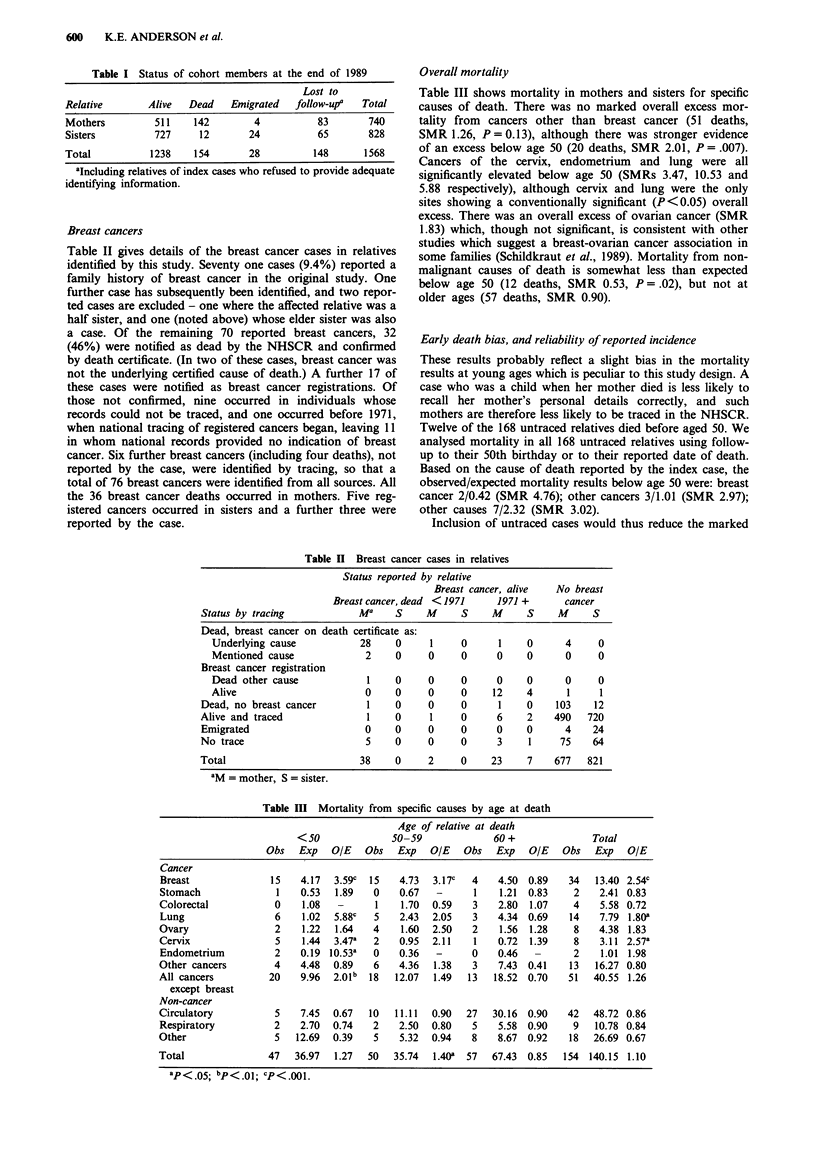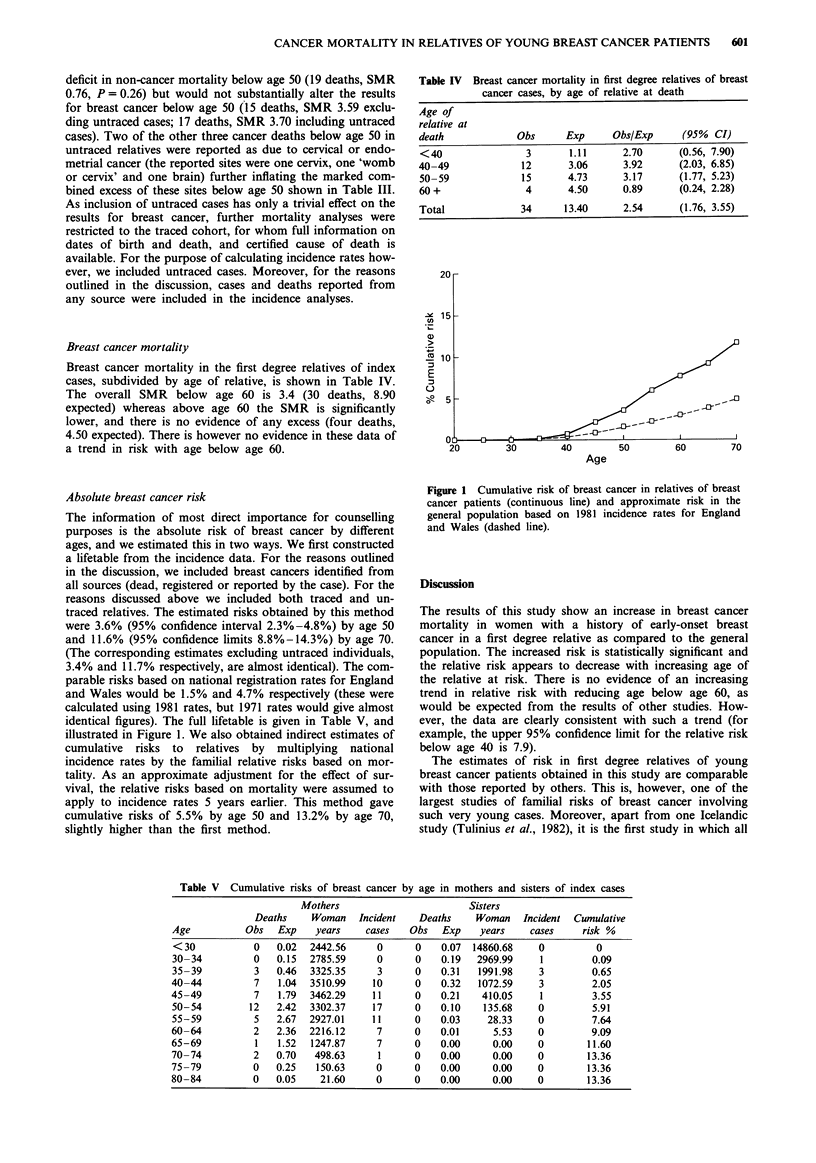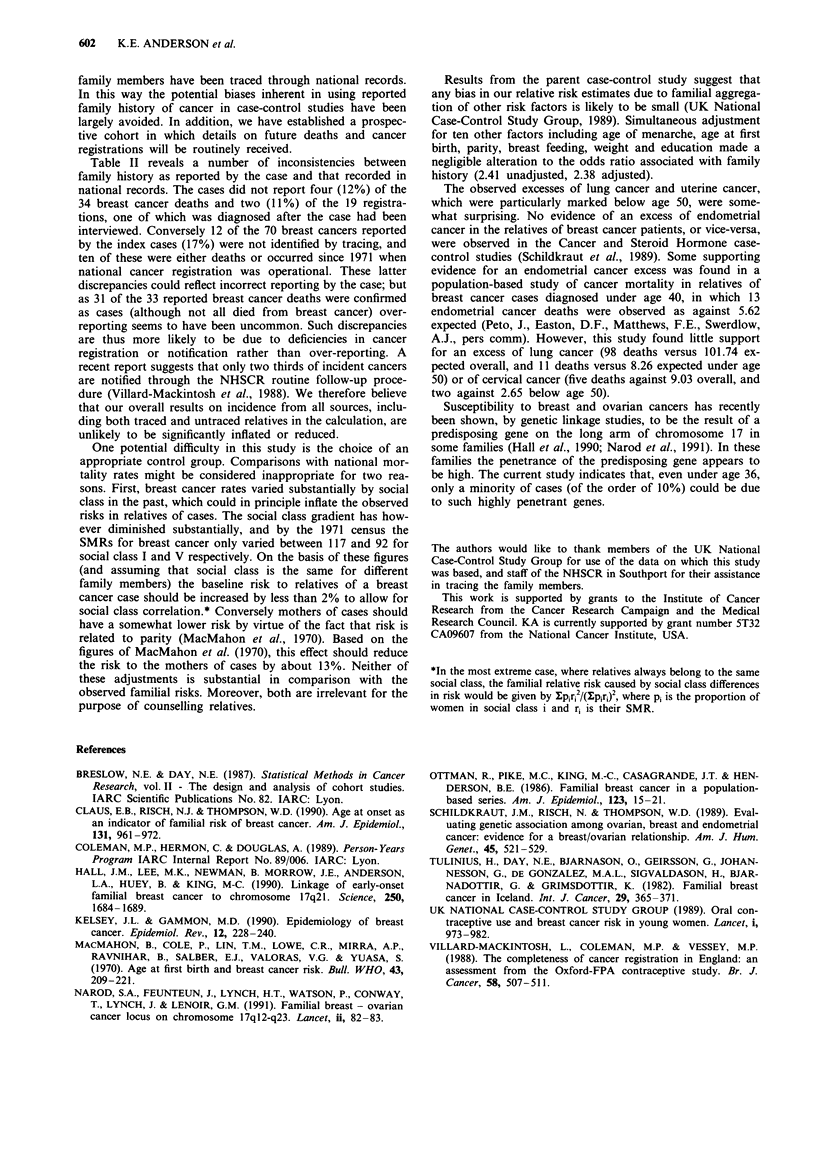# Cancer mortality in the first degree relatives of young breast cancer patients.

**DOI:** 10.1038/bjc.1992.321

**Published:** 1992-09

**Authors:** K. E. Anderson, D. F. Easton, F. E. Matthews, J. Peto

**Affiliations:** Division of Epidemiology, School of Public Health, University of Minnesota, Minneapolis.

## Abstract

In a retrospective cohort study, the mothers and sisters of 740 breast cancer patients aged under 36 at diagnosis have been studied for mortality and cancer incidence. Significantly increased breast cancer mortality was observed below age 60 (30 deaths; SMR = 3.4), but not at older ages (four deaths; SMR = 0.9). The cumulative breast cancer incidence in the relatives was 3.6% by age 50, 7.6% by age 60 and 11.6% by age 70. They also suffered excess mortality below age 60 for cancers of reproductive sites (cervix, ovary and endometrium; 15 deaths; SMR = 2.6) and lung (11 deaths; SMR = 3.2), but not for other sites (12 deaths; SMR = 0.9). This large population-based cohort study provides further confirmation of genetic susceptibility to breast cancer at young ages.


Br. J. Cancer (1992), 66, 599-602 © Macmillan Press Ltd., 1992

### Cancer mortality in the first degree relatives of young breast cancer patients

K.E. Anderson[1], D.F. Easton[2], F.E. Matthews[2] & J. Peto[2]

[1]*Division of Epidemiology, School of Public Health, University of Minnesota, Minneapolis, Minnesota;* [2]*Section of Epidemiology, Institute of Cancer Research, Sutton, Surrey.*

**Summary** In a retrospective cohort study, the mothers and sisters of 740 breast cancer patients aged under 36 at diagnosis have been studied for mortality and cancer incidence. Significantly increased breast cancer mortality was observed below age 60 (30 deaths; SMR = 3.4), but not at older ages (four deaths; SMR = 0.9). The cumulative breast cancer incidence in the relatives was 3.6% by age 50, 7.6% by age 60 and 11.6% by age 70. They also suffered excess mortality below age 60 for cancers of reproductive sites (cervix, ovary and endometrium; 15 deaths; SMR = 2.6) and lung (11 deaths; SMR = 3.2), but not for other sites (12 deaths; SMR = 0.9). This large population-based cohort study provides further confirmation of genetic susceptibility to breast cancer at young ages.

A family history of breast cancer in a close relative is a well known risk factor for the disease (Kelsey & Gammon, 1990). Various studies have shown that the risk in first degree relatives is greatest for those with a family history of breast cancer with an early age of onset (Ottman *et al.*, 1986; Claus *et al.*, 1990). Most such studies are, however, based to some extent on family history as reported by the index case, although cases may have been confirmed by hospital records or death certificates in some studies. Unbiased data on the magnitude of the risk, and on its dependence on age at onset of the index case and her relatives, are needed both for genetic counselling and to elucidate the underlying mechanisms of breast cancer.

In this study, 740 index cases were identified in a population-based case–control study of breast cancer and oral contraceptive use (UK National Case-Control Study Group, 1989). Details provided by the cases were used to trace their mothers and sisters through the National Health Service Central Register (NHSCR) for mortality and cancer incidence. This tracing procedure provides almost unbiased estimates of mortality and cancer incidence among relatives compared with national rates, and also establishes a prospective cohort for future analysis.

### Methods

### *Case selection and interviewing*

The methods of case ascertainment and data collection have been fully described elsewhere (UK National Case-Control Study Group, 1989). All known cases of breast cancer in women aged under 36 diagnosed between January 1, 1982 and December 31, 1985 who were resident in 11 regional health authority areas in Britain were ascertained. Cases were identified primarily through local cancer registries, with additional information from hospital discharge and computerised patient lists at major treatment centres. The study was restricted to white women diagnosed in Britain with no previous malignancy, severe mental handicap or psychiatric condition. All diagnoses were confirmed by pathology report. After obtaining permission from the responsible clinician, the cases were contacted by letter and subsequent telephone call, and were invited to participate in a 'study of women's health'. Cases who initially refused were contacted again 6 months later. Of the total of 1049 cases diagnosed in the study area and period, 16% had died prior to contact, and the clinician refused permission to contact a further 7%. The overall response rate among the remaining 811 cases was 90%. Women who agreed to take part were visited by trained interviewers in their homes.

### *Family tracing*

During the routine face to face interview, the cases were asked to provide full names and dates of birth of their mother and any sisters. Only full sisters and natural mothers were included in the cohort.

The mothers and sisters were traced through the NHSCR to obtain details of deaths and cancer registrations. The Department of Health and Social Security Register was also used to trace individuals whose records were not found in the NHSCR. Follow-up for the entire cohort is complete to the end of 1989. Deaths were coded according to the ninth revision of the International Classification of Diseases (ICD).

### *Statistical methods*

As incident cancers were not recorded in the NHSCR before 1971 and registration is not complete thereafter, relative risk estimates are based only on the mortality data. Woman-years at risk were calculated using the 'Person-Years' program, (Coleman *et al.*, 1989). Follow-up began on their 10th birthday for sisters, and on the date of birth of the case for mothers. Deaths and woman-years after age 85 were ignored. Follow-up closed on December 31, 1989. Expected numbers of deaths were computed using age-, sex- and calendar period-specific mortality rates for England and Wales. Two-sided 95% confidence intervals for relative risk estimates are based on the Poisson distribution (Breslow & Day, 1987).

### Results

### *Follow-up*

Of the 755 cases in the original study we excluded 14 who were adopted, and one whose sister was also a case. (To avoid double counting only the elder sister was included.)

The follow up status of the relatives of the remaining 740 cases is shown in Table 1. Of the 1568 first degree female relatives, 2% (28) had emigrated and 9% (148) were either untraced or had never lived in Britain. One hundred and fifty four had died before the end of 1989, and 1238 were alive.

Correspondence: J. Peto, Section of Epidemiology, Block 'D', Institute of Cancer Research, 15 Cotswold Road, Belmont, Surrey, SM2 5NG, UK.
Received and accepted 26 November 1991.

**Table I** Status of cohort members at the end of 1989

| *Relative* | *Alive* | *Dead* | *Emigrated* | *Lost to follow-up*[a] | *Total* |
|---|---|---|---|---|---|
| Mothers | 511 | 142 | 4 | 83 | 740 |
| Sisters | 727 | 12 | 24 | 65 | 828 |
| Total | 1238 | 154 | 28 | 148 | 1568 |

[a]Including relatives of index cases who refused to provide adequate identifying information.

### *Breast cancers*

Table II gives details of the breast cancer cases in relatives identified by this study. Seventy one cases (9.4%) reported a family history of breast cancer in the original study. One further case has subsequently been identified, and two reported cases are excluded – one where the affected relative was a half sister, and one (noted above) whose elder sister was also a case. Of the remaining 70 reported breast cancers, 32 (46%) were notified as dead by the NHSCR and confirmed by death certificate. (In two of these cases, breast cancer was not the underlying certified cause of death.) A further 17 of these cases were notified as breast cancer registrations. Of those not confirmed, nine occurred in individuals whose records could not be traced, and one occurred before 1971, when national tracing of registered cancers began, leaving 11 in whom national records provided no indication of breast cancer. Six further breast cancers (including four deaths), not reported by the case, were identified by tracing, so that a total of 76 breast cancers were identified from all sources. All the 36 breast cancer deaths occurred in mothers. Five registered cancers occurred in sisters and a further three were reported by the case.

### *Overall mortality*

Table III shows mortality in mothers and sisters for specific causes of death. There was no marked overall excess mortality from cancers other than breast cancer (51 deaths, SMR 1.26, $P = 0.13$), although there was stronger evidence of an excess below age 50 (20 deaths, SMR 2.01, $P = .007$). Cancers of the cervix, endometrium and lung were all significantly elevated below age 50 (SMRs 3.47, 10.53 and 5.88 respectively), although cervix and lung were the only sites showing a conventionally significant ($P < 0.05$) overall excess. There was an overall excess of ovarian cancer (SMR 1.83) which, though not significant, is consistent with other studies which suggest a breast-ovarian cancer association in some families (Schildkraut *et al.*, 1989). Mortality from non-malignant causes of death is somewhat less than expected below age 50 (12 deaths, SMR 0.53, $P = .02$), but not at older ages (57 deaths, SMR 0.90).

### *Early death bias, and reliability of reported incidence*

These results probably reflect a slight bias in the mortality results at young ages which is peculiar to this study design. A case who was a child when her mother died is less likely to recall her mother's personal details correctly, and such mothers are therefore less likely to be traced in the NHSCR. Twelve of the 168 untraced relatives died before aged 50. We analysed mortality in all 168 untraced relatives using follow-up to their 50th birthday or to their reported date of death. Based on the cause of death reported by the index case, the observed/expected mortality results below age 50 were: breast cancer 2/0.42 (SMR 4.76); other cancers 3/1.01 (SMR 2.97); other causes 7/2.32 (SMR 3.02).

Inclusion of untraced cases would thus reduce the marked

**Table II** Breast cancer cases in relatives

| | *Status reported by relative* | | | | | | | |
|---|---|---|---|---|---|---|---|---|
| | *Breast cancer, dead* | | *Breast cancer, alive* *<1971* | | *1971+* | | *No breast cancer* | |
| *Status by tracing* | *M*[a] | *S* | *M* | *S* | *M* | *S* | *M* | *S* |
| Dead, breast cancer on death certificate as: | | | | | | | | |
| Underlying cause | 28 | 0 | 1 | 0 | 1 | 0 | 4 | 0 |
| Mentioned cause | 2 | 0 | 0 | 0 | 0 | 0 | 0 | 0 |
| Breast cancer registration | | | | | | | | |
| Dead other cause | 1 | 0 | 0 | 0 | 0 | 0 | 0 | 0 |
| Alive | 0 | 0 | 0 | 0 | 12 | 4 | 1 | 1 |
| Dead, no breast cancer | 1 | 0 | 0 | 0 | 1 | 0 | 103 | 12 |
| Alive and traced | 1 | 0 | 1 | 0 | 6 | 2 | 490 | 720 |
| Emigrated | 0 | 0 | 0 | 0 | 0 | 0 | 4 | 24 |
| No trace | 5 | 0 | 0 | 0 | 3 | 1 | 75 | 64 |
| Total | 38 | 0 | 2 | 0 | 23 | 7 | 677 | 821 |

[a]M = mother, S = sister.

**Table III** Mortality from specific causes by age at death

| | *Age of relative at death* *<50* | | | *50–59* | | | *60+* | | | *Total* | | |
|---|---|---|---|---|---|---|---|---|---|---|---|---|
| | *Obs* | *Exp* | *O/E* | *Obs* | *Exp* | *O/E* | *Obs* | *Exp* | *O/E* | *Obs* | *Exp* | *O/E* |
| *Cancer* | | | | | | | | | | | | |
| Breast | 15 | 4.17 | 3.59[c] | 15 | 4.73 | 3.17[c] | 4 | 4.50 | 0.89 | 34 | 13.40 | 2.54[c] |
| Stomach | 1 | 0.53 | 1.89 | 0 | 0.67 | – | 1 | 1.21 | 0.83 | 2 | 2.41 | 0.83 |
| Colorectal | 0 | 1.08 | – | 1 | 1.70 | 0.59 | 3 | 2.80 | 1.07 | 4 | 5.58 | 0.72 |
| Lung | 6 | 1.02 | 5.88[c] | 5 | 2.43 | 2.05 | 3 | 4.34 | 0.69 | 14 | 7.79 | 1.80[a] |
| Ovary | 2 | 1.22 | 1.64 | 4 | 1.60 | 2.50 | 2 | 1.56 | 1.28 | 8 | 4.38 | 1.83 |
| Cervix | 5 | 1.44 | 3.47[a] | 2 | 0.95 | 2.11 | 1 | 0.72 | 1.39 | 8 | 3.11 | 2.57[a] |
| Endometrium | 2 | 0.19 | 10.53[a] | 0 | 0.36 | – | 0 | 0.46 | – | 2 | 1.01 | 1.98 |
| Other cancers | 4 | 4.48 | 0.89 | 6 | 4.36 | 1.38 | 3 | 7.43 | 0.41 | 13 | 16.27 | 0.80 |
| All cancers except breast | 20 | 9.96 | 2.01[b] | 18 | 12.07 | 1.49 | 13 | 18.52 | 0.70 | 51 | 40.55 | 1.26 |
| *Non-cancer* | | | | | | | | | | | | |
| Circulatory | 5 | 7.45 | 0.67 | 10 | 11.11 | 0.90 | 27 | 30.16 | 0.90 | 42 | 48.72 | 0.86 |
| Respiratory | 2 | 2.70 | 0.74 | 2 | 2.50 | 0.80 | 5 | 5.58 | 0.90 | 9 | 10.78 | 0.84 |
| Other | 5 | 12.69 | 0.39 | 5 | 5.32 | 0.94 | 8 | 8.67 | 0.92 | 18 | 26.69 | 0.67 |
| Total | 47 | 36.97 | 1.27 | 50 | 35.74 | 1.40[a] | 57 | 67.43 | 0.85 | 154 | 140.15 | 1.10 |

[a]$P < .05$; [b]$P < .01$; [c]$P < .001$.

deficit in non-cancer mortality below age 50 (19 deaths, SMR 0.76, $P = 0.26$) but would not substantially alter the results for breast cancer below age 50 (15 deaths, SMR 3.59 excluding untraced cases; 17 deaths, SMR 3.70 including untraced cases). Two of the other three cancer deaths below age 50 in untraced relatives were reported as due to cervical or endometrial cancer (the reported sites were one cervix, one 'womb or cervix' and one brain) further inflating the marked combined excess of these sites below age 50 shown in Table III. As inclusion of untraced cases has only a trivial effect on the results for breast cancer, further mortality analyses were restricted to the traced cohort, for whom full information on dates of birth and death, and certified cause of death is available. For the purpose of calculating incidence rates however, we included untraced cases. Moreover, for the reasons outlined in the discussion, cases and deaths reported from any source were included in the incidence analyses.

### *Breast cancer mortality*

Breast cancer mortality in the first degree relatives of index cases, subdivided by age of relative, is shown in Table IV. The overall SMR below age 60 is 3.4 (30 deaths, 8.90 expected) whereas above age 60 the SMR is significantly lower, and there is no evidence of any excess (four deaths, 4.50 expected). There is however no evidence in these data of a trend in risk with age below age 60.

### *Absolute breast cancer risk*

The information of most direct importance for counselling purposes is the absolute risk of breast cancer by different ages, and we estimated this in two ways. We first constructed a lifetable from the incidence data. For the reasons outlined in the discussion, we included breast cancers identified from all sources (dead, registered or reported by the case). For the reasons discussed above we included both traced and untraced relatives. The estimated risks obtained by this method were 3.6% (95% confidence interval 2.3%–4.8%) by age 50 and 11.6% (95% confidence limits 8.8%–14.3%) by age 70. (The corresponding estimates excluding untraced individuals, 3.4% and 11.7% respectively, are almost identical). The comparable risks based on national registration rates for England and Wales would be 1.5% and 4.7% respectively (these were calculated using 1981 rates, but 1971 rates would give almost identical figures). The full lifetable is given in Table V, and illustrated in Figure 1. We also obtained indirect estimates of cumulative risks to relatives by multiplying national incidence rates by the familial relative risks based on mortality. As an approximate adjustment for the effect of survival, the relative risks based on mortality were assumed to apply to incidence rates 5 years earlier. This method gave cumulative risks of 5.5% by age 50 and 13.2% by age 70, slightly higher than the first method.

**Table IV** Breast cancer mortality in first degree relatives of breast cancer cases, by age of relative at death

| *Age of relative at death* | *Obs* | *Exp* | *Obs/Exp* | *(95% CI)* |
|---|---|---|---|---|
| <40 | 3 | 1.11 | 2.70 | (0.56, 7.90) |
| 40–49 | 12 | 3.06 | 3.92 | (2.03, 6.85) |
| 50–59 | 15 | 4.73 | 3.17 | (1.77, 5.23) |
| 60 + | 4 | 4.50 | 0.89 | (0.24, 2.28) |
| Total | 34 | 13.40 | 2.54 | (1.76, 3.55) |

**Figure 1** Cumulative risk of breast cancer in relatives of breast cancer patients (continuous line) and approximate risk in the general population based on 1981 incidence rates for England and Wales (dashed line).

### Discussion

The results of this study show an increase in breast cancer mortality in women with a history of early-onset breast cancer in a first degree relative as compared to the general population. The increased risk is statistically significant and the relative risk appears to decrease with increasing age of the relative at risk. There is no evidence of an increasing trend in relative risk with reducing age below age 60, as would be expected from the results of other studies. However, the data are clearly consistent with such a trend (for example, the upper 95% confidence limit for the relative risk below age 40 is 7.9).

The estimates of risk in first degree relatives of young breast cancer patients obtained in this study are comparable with those reported by others. This is, however, one of the largest studies of familial risks of breast cancer involving such very young cases. Moreover, apart from one Icelandic study (Tulinius *et al.*, 1982), it is the first study in which all

**Table V** Cumulative risks of breast cancer by age in mothers and sisters of index cases

| | *Mothers* | | | | *Sisters* | | | | |
|---|---|---|---|---|---|---|---|---|---|
| | *Deaths* | | *Woman* | *Incident* | *Deaths* | | *Woman* | *Incident* | *Cumulative* |
| *Age* | *Obs* | *Exp* | *years* | *cases* | *Obs* | *Exp* | *years* | *cases* | *risk %* |
| <30 | 0 | 0.02 | 2442.56 | 0 | 0 | 0.07 | 14860.68 | 0 | 0 |
| 30–34 | 0 | 0.15 | 2785.59 | 0 | 0 | 0.19 | 2969.99 | 1 | 0.09 |
| 35–39 | 3 | 0.46 | 3325.35 | 3 | 0 | 0.31 | 1991.98 | 3 | 0.65 |
| 40–44 | 7 | 1.04 | 3510.99 | 10 | 0 | 0.32 | 1072.59 | 3 | 2.05 |
| 45–49 | 7 | 1.79 | 3462.29 | 11 | 0 | 0.21 | 410.05 | 1 | 3.55 |
| 50–54 | 12 | 2.42 | 3302.37 | 17 | 0 | 0.10 | 135.68 | 0 | 5.91 |
| 55–59 | 5 | 2.67 | 2927.01 | 11 | 0 | 0.03 | 28.33 | 0 | 7.64 |
| 60–64 | 2 | 2.36 | 2216.12 | 7 | 0 | 0.01 | 5.53 | 0 | 9.09 |
| 65–69 | 1 | 1.52 | 1247.87 | 7 | 0 | 0.00 | 0.00 | 0 | 11.60 |
| 70–74 | 2 | 0.70 | 498.63 | 1 | 0 | 0.00 | 0.00 | 0 | 13.36 |
| 75–79 | 0 | 0.25 | 150.63 | 0 | 0 | 0.00 | 0.00 | 0 | 13.36 |
| 80–84 | 0 | 0.05 | 21.60 | 0 | 0 | 0.00 | 0.00 | 0 | 13.36 |

family members have been traced through national records. In this way the potential biases inherent in using reported family history of cancer in case-control studies have been largely avoided. In addition, we have established a prospective cohort in which details on future deaths and cancer registrations will be routinely received.

Table II reveals a number of inconsistencies between family history as reported by the case and that recorded in national records. The cases did not report four (12%) of the 34 breast cancer deaths and two (11%) of the 19 registrations, one of which was diagnosed after the case had been interviewed. Conversely 12 of the 70 breast cancers reported by the index cases (17%) were not identified by tracing, and ten of these were either deaths or occurred since 1971 when national cancer registration was operational. These latter discrepancies could reflect incorrect reporting by the case; but as 31 of the 33 reported breast cancer deaths were confirmed as cases (although not all died from breast cancer) over-reporting seems to have been uncommon. Such discrepancies are thus more likely to be due to deficiencies in cancer registration or notification rather than over-reporting. A recent report suggests that only two thirds of incident cancers are notified through the NHSCR routine follow-up procedure (Villard-Mackintosh *et al.*, 1988). We therefore believe that our overall results on incidence from all sources, including both traced and untraced relatives in the calculation, are unlikely to be significantly inflated or reduced.

One potential difficulty in this study is the choice of an appropriate control group. Comparisons with national mortality rates might be considered inappropriate for two reasons. First, breast cancer rates varied substantially by social class in the past, which could in principle inflate the observed risks in relatives of cases. The social class gradient has however diminished substantially, and by the 1971 census the SMRs for breast cancer only varied between 117 and 92 for social class I and V respectively. On the basis of these figures (and assuming that social class is the same for different family members) the baseline risk to relatives of a breast cancer case should be increased by less than 2% to allow for social class correlation.* Conversely mothers of cases should have a somewhat lower risk by virtue of the fact that risk is related to parity (MacMahon *et al.*, 1970). Based on the figures of MacMahon *et al.* (1970), this effect should reduce the risk to the mothers of cases by about 13%. Neither of these adjustments is substantial in comparison with the observed familial risks. Moreover, both are irrelevant for the purpose of counselling relatives.

Results from the parent case-control study suggest that any bias in our relative risk estimates due to familial aggregation of other risk factors is likely to be small (UK National Case-Control Study Group, 1989). Simultaneous adjustment for ten other factors including age of menarche, age at first birth, parity, breast feeding, weight and education made a negligible alteration to the odds ratio associated with family history (2.41 unadjusted, 2.38 adjusted).

The observed excesses of lung cancer and uterine cancer, which were particularly marked below age 50, were somewhat surprising. No evidence of an excess of endometrial cancer in the relatives of breast cancer patients, or vice-versa, were observed in the Cancer and Steroid Hormone case-control studies (Schildkraut *et al.*, 1989). Some supporting evidence for an endometrial cancer excess was found in a population-based study of cancer mortality in relatives of breast cancer cases diagnosed under age 40, in which 13 endometrial cancer deaths were observed as against 5.62 expected (Peto, J., Easton, D.F., Matthews, F.E., Swerdlow, A.J., pers comm). However, this study found little support for an excess of lung cancer (98 deaths versus 101.74 expected overall, and 11 deaths versus 8.26 expected under age 50) or of cervical cancer (five deaths against 9.03 overall, and two against 2.65 below age 50).

Susceptibility to breast and ovarian cancers has recently been shown, by genetic linkage studies, to be the result of a predisposing gene on the long arm of chromosome 17 in some families (Hall *et al.*, 1990; Narod *et al.*, 1991). In these families the penetrance of the predisposing gene appears to be high. The current study indicates that, even under age 36, only a minority of cases (of the order of 10%) could be due to such highly penetrant genes.

The authors would like to thank members of the UK National Case-Control Study Group for use of the data on which this study was based, and staff of the NHSCR in Southport for their assistance in tracing the family members.

This work is supported by grants to the Institute of Cancer Research from the Cancer Research Campaign and the Medical Research Council. KA is currently supported by grant number 5T32 CA09607 from the National Cancer Institute, USA.

*In the most extreme case, where relatives always belong to the same social class, the familial relative risk caused by social class differences in risk would be given by $\Sigma p_i r_i^2/(\Sigma p_i r_i)^2$, where $p_i$ is the proportion of women in social class i and $r_i$ is their SMR.

### References

BRESLOW, N.E. & DAY, N.E. (1987). *Statistical Methods in Cancer Research*, vol. II - The design and analysis of cohort studies. IARC Scientific Publications No. 82. IARC: Lyon.

CLAUS, E.B., RISCH, N.J. & THOMPSON, W.D. (1990). Age at onset as an indicator of familial risk of breast cancer. *Am. J. Epidemiol.*, **131**, 961-972.

COLEMAN, M.P., HERMON, C. & DOUGLAS, A. (1989). *Person-Years Program* IARC Internal Report No. 89/006. IARC: Lyon.

HALL, J.M., LEE, M.K., NEWMAN, B. MORROW, J.E., ANDERSON, L.A., HUEY, B. & KING, M-C. (1990). Linkage of early-onset familial breast cancer to chromosome 17q21. *Science*, **250**, 1684-1689.

KELSEY, J.L. & GAMMON, M.D. (1990). Epidemiology of breast cancer. *Epidemiol. Rev.*, **12**, 228-240.

MACMAHON, B., COLE, P., LIN, T.M., LOWE, C.R., MIRRA, A.P., RAVNIHAR, B., SALBER, E.J., VALORAS, V.G. & YUASA, S. (1970). Age at first birth and breast cancer risk. *Bull. WHO*, **43**, 209-221.

NAROD, S.A., FEUNTEUN, J., LYNCH, H.T., WATSON, P., CONWAY, T., LYNCH, J. & LENOIR, G.M. (1991). Familial breast - ovarian cancer locus on chromosome 17q12-q23. *Lancet*, **ii**, 82-83.

OTTMAN, R., PIKE, M.C., KING, M.-C., CASAGRANDE, J.T. & HENDERSON, B.E. (1986). Familial breast cancer in a population-based series. *Am. J. Epidemiol.*, **123**, 15-21.

SCHILDKRAUT, J.M., RISCH, N. & THOMPSON, W.D. (1989). Evaluating genetic association among ovarian, breast and endometrial cancer: evidence for a breast/ovarian relationship. *Am. J. Hum. Genet.*, **45**, 521-529.

TULINIUS, H., DAY, N.E., BJARNASON, O., GEIRSSON, G., JOHANNESSON, G., DE GONZALEZ, M.A.L., SIGVALDASON, H., BJARNADOTTIR, G. & GRIMSDOTTIR, K. (1982). Familial breast cancer in Iceland. *Int. J. Cancer*, **29**, 365-371.

UK NATIONAL CASE-CONTROL STUDY GROUP (1989). Oral contraceptive use and breast cancer risk in young women. *Lancet*, **i**, 973-982.

VILLARD-MACKINTOSH, L., COLEMAN, M.P. & VESSEY, M.P. (1988). The completeness of cancer registration in England: an assessment from the Oxford-FPA contraceptive study. *Br. J. Cancer*, **58**, 507-511.